# Green Synthesis of an Activated Carbon-Supported Ag and ZnO Nanocomposite for Photocatalytic Degradation and Its Antibacterial Activities

**DOI:** 10.3390/molecules25071586

**Published:** 2020-03-30

**Authors:** Amel Taha, Melek Ben Aissa, Enshirah Da’na

**Affiliations:** 1 Department of Chemistry, King Faisal University, Alahsa 31982, Saudi Arabia; ataha@kfu.edu.sa; 2 Department of Chemistry, Faculty of Science and Technology, Al-Neelain University, Khartoum 11121, Sudan; 3 Community College in Albuqaiq, King Faisal University, 31992, Saudi Arabia; maissa@kfu.edu.sa; 4 Biomedical Engineering Department, King Faisal University, Alahsa 31982, Saudi Arabia

**Keywords:** nanocomposite, green synthesis, activated carbon, antibacterial, catalytic activity, neem leaf extract

## Abstract

In this study Ag nanoparticles (AgNPs), ZnO nanoparticles (ZnONPs), and Ag/ZnO nanocomposites were greenly synthesized and loaded on activated carbon via three different routes: simple impregnation, successive precipitation, and co-precipitation. Neem leaf extract was used as a reducing and stabilizing agent. The morphological and structural properties of the synthesized nanocomposites have been examined using different analytical techniques such as XRD, SEM, FTIR, and UV. The antibacterial and catalytic activity of the synthesized nanocomposites were examined and compared. The results showed that AgNPs loaded on activated carbon (Ag/AC) has the best catalytic activity compared to the other nanocomposites, which is attributed to the good dispersal of AgNPs on the surface of activated carbon. Furthermore, AgNPs showed the best antibacterial effect on eight out of 16 tested pathogens. Results also showed that the order of precipitation is an important factor, as both antibacterial activities and photodegradation activities were higher for ZnO/Ag/AC than Ag/ZnO/AC. Furthermore, the co-precipitation method was shown to be better than the successive precipitation method for 4-nitrophenol photodegradation and 14 out of the 16 antibacterial tests performed.

## 1. Introduction

General sanitization, especially for infections caused by water, is a severe health problem. Threats can be physical, chemical, or microbiological causes of infection. Activated carbon (AC) is commonly utilized for water purification due to its huge surface area, which results in high adsorption efficiency. Also, AC has non-localized n electrons and is also rich with functional groups such as COOH, OH, NH_2_ and amide, which attract different species via hydrogen bonds and electrostatic forces [1]. However, for activated carbon to remove both chemical and microbiological problems, a special functionalization should be done. Activated carbon in its bare form supports the growth of bacteria due to its biocompatibility. Activated carbon with antibacterial activity can be produced by impregnation of silver nanoparticles [2,3,4,5,6], or metallic oxides such as ZnO [7]. Furthermore, silver and ZnO nanoparticles supported on activated carbon have gained much attention for adsorption [6,8,9,10,11,12,13,14,15,16], and catalytic applications [7,17,18,19,20,21,22].

4-Nitrophenol is extensively used in many industries to make explosives, wood protection products, dyes, and herbicides. 4-Nitrophenol is considered as a major pollutant due to its high solubility and stability in water [23]. Recently, the degradation of 4-nitrophenol has become an essential topic since it produces an extremely useful intermediate, 4-aminophenol, which is widely used in the synthesis of drugs such as paracetamol and acetaminophen [24]. One of the most effective method for the reduction of the toxic 4-nitrophenol into useful 4-aminophenol is by catalytic reduction. This method has been considered as a green process since it does not require any organic solvent [24]. Accordingly, the development of a suitable catalyst for this process would be vital for environment protection and for pharmaceutical industry.

AgNPs has been reported to have a high reduction activity toward 4-nitrophenol due to the facilitated electron transfer [24]. However, aggregation of AgNPs minimizes the surface area of catalysts and reduces the catalytic activity of the particles. A reasonable solution to overcome this problem is to immobilize the AgNPs onto a support such as activated carbon [25].

Recently, metal oxides such as ZnO have attracted much attention in catalytic degradation of toxic organic pollutants. This is mainly because it has high efficiency, low cost, thermal and chemical stability, non-toxicity, and environmental-friendly nature [12,22,26]. On the other hand, it has several drawbacks such as low quantum yield, the high recombination rate of photo-induced hole-electron pairs, and instability [20,27], which hinder its commercial applications [10,21,28,29]. To overcome these drawbacks of ZnO, many different approaches have been developed such as metal doping of ZnO [30,31,32,33,34], organic modification of the surface, and semiconductor coupling [35].

It was reported that the TiO_2_/ZnO hybrid nanocomposite shows an improved photocatalytic activity toward many organic pollutants compared to that of ZnO or TiO_2_ alone [20]. ZnO/CuO nanocomposite has also been applied for its photocatalytic activity [34]. ZnO/MgO nanocomposite has been reported to have much higher catalyst photoactivity when compared with the ZnO [10].

Recently, many efforts have been spent on doping ZnO with noble metals to produce nanocomposite structures. Cerium has been used as dopant in ZnO to increase its photocatalytic activity because it acts as an electron reservoir [27]. The most applied metal/ZnO nanocomposite is Ag/ZnO [11,19,26,30,31,35,36], due to the Ag nanoparticles antibacterial [37], and catalytic activity since it has a high surface area [9,24]. Furthermore, Ag is recognized as an electron sink.

So far, several approaches have been described for the synthesize Ag/ZnO nanocomposites including sol-gel, photoreduction, chemical deposition, hydrothermal, and pulsed laser deposition [26]. However, most of these approaches cannot be commercially applied since they require high temperatures, high pressures, toxic chemicals, expensive equipment, or long synthesis times. Thus, a cheap, safe, and simple approach for Ag/ZnO nanocomposites is essential to meet environmental and economic needs. Although significant efforts have been spent in the synthesis of Ag/ZnO nanocomposite, only a few pieces of this research have focused on green synthesis approaches [11].

The need for environmentally friendly synthetic procedures for nanoparticles leads to a high demand for green nanotechnology [38,39,40,41,42,43]. Many parts of plants such as leaf, fruit, stem, bark, root, leaf, and bud have been utilized for the green synthesis of Ag nanoparticles. Many plant products such as *Azadirachta indica* (Neem) leaf extract [38,44,45]. *Tribulus longipetalus* extract [40], *Acacia nilotica* pods [46], *Ficus altissima* Blume leaf extract [47], and aqueous leaf extracts of *Emblica officinalis* [42], are capable of reducing metal ions to nanoparticles because of their antioxidant properties. Furthermore, plant extracts produce capping agents for the stabilization of nanoparticles.

Neem plant, scientifically known as *Azadirachta indica*, is found plentifully in Saudi Arabia and is related to the Meliaceae family. It has many medicinal properties, which make it useful for various medical applications. Furthermore, it contains two important classes of chemicals, namely terpenoids and flavanones. These compounds act as reducing as well as a capping agents to stabilize nanoparticles [45,48,49].

Accordingly, the main objective of this work was to combine green synthesis, metal doping, and hybrid nanocomposites for the synthesis of nanocomposites by three different routes: simple impregnation (Ag/AC and ZnO/AC), co-precipitation (Ag-ZnO/AC), and successive precipitation (Ag/ZnO/AC and ZnO/Ag/AC) and to investigate the structural properties, antibacterial efficiency against MDR pathogenic bacteria, and catalytic activity toward photodegradation of 4-nitrophenol of the resulting products in an attempt to provide an approach that can yield affordable products for water treatment purposes. For this purpose, neem leaf extract was used as a reducing agent and stabilizer of Ag and ZnO nanoparticles (NPs). Furthermore, to support the green synthesis route, the activated carbon used in this work was obtained from spent activated carbon used in home filtration systems.

## 2. Results and Discussion

Green synthesis eliminates the use or generation of hazardous substances and reduces the consumption of non-renewable resources. In this work, the two concepts were achieved by recycling and reusing of a spent activated carbon as a support instead of using fresh material. This was achieved by collecting disposed activated carbon used in home filtration systems and regenerating it. Furthermore, the reduction and stabilization of the nanoparticles were achieved by using neem leaf extract as a reducing and capping agent. Accordingly, the need for reducing and stabilizing chemicals was eliminated [2,44]. Also, the application of the developed nanocomposites in catalytic reduction of 4-nitrophenol into useful 4-aminophenol is considered as a green process since it does not require any organic solvent [24].

**Figure 1:**
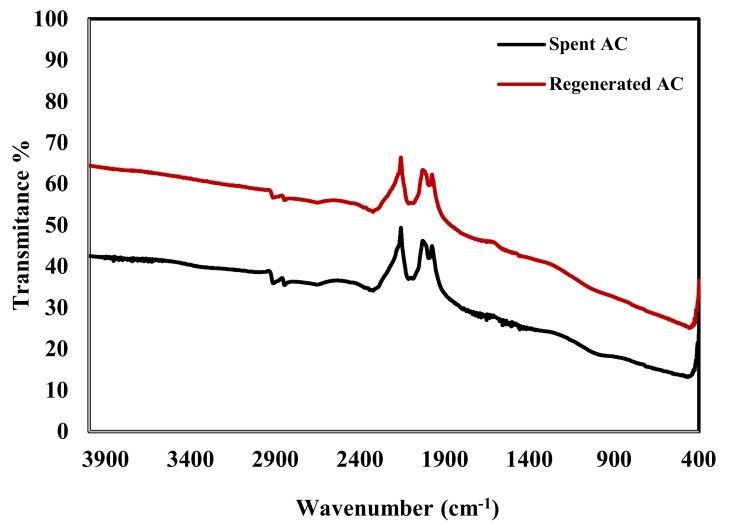
FTIR spectrum of the spent and regenerated activated carbons.

Figure 1 shows that after regeneration of the spent activated carbon it still contains the main bands that enhance the adsorption of NPs on AC. The bands detected at 2962 cm^−1^, 1625 cm^−1^, 1564 cm^−1^, 2320 cm^−1^, and 1000 cm^−1^ are related to -CH stretching, C=C aromatic stretching, C=O stretching of carbonyl group, hydrogen-bonded OH, and C–O stretching and OH bending of alcohol and carboxylic acids, respectively. The good adsorption tendency of AC towards NPs is related to the existence of these functional groups on its surface [50].

**Figure 2:**
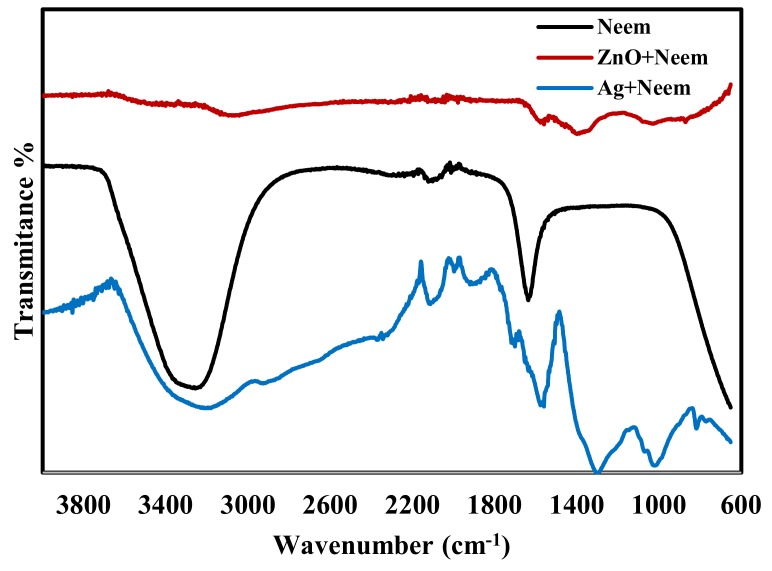
FTIR spectra of pure neem leaf extract, Neem extract mixed with Zn(NO_3_)_2_ solution and neem extract mixed with AgNO_3_ solution.

Figure 2 displays the FTIR spectra of pure neem leaf extract. The strong stretching band that appears around 3239 cm^−1^ is related to the overlapping of the bending vibration of N–H of the amine group and the OH stretching vibration of the phenolic group in the neem leaf extract. The OH groups in phenols have been reported as a reduction agent for Ag^+^ into Ag and Zn^+2^ into ZnO [51]. After mixing the neem leaf extract with AgNO_3_ solution there was a decrease in the strength of the peak at 3433 cm^−1^ implying that phenolic groups of neem are involved in the reduction reaction [49]. A similar observation was made when neem extract was mixed with Zn(NO_3_)_2_ solution, there was a shift with a huge decrease in the intensity of the OH group. The FTIR spectra in Figure 2 also shows a peak at 1636 cm^−1^, which is related to C=O stretching of -COOH group in the neem leaf extract. Many studies have reported that C=O−C and C=O groups can act as a stabilizing agent [51].

**Figure 3:**
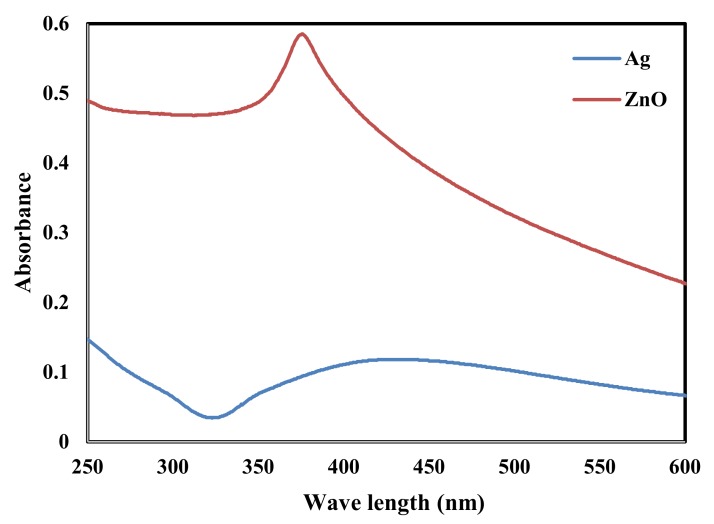
UV-Vis spectra of AgNPs and ZnONPs.

The addition of the neem leaf extract to the colorless AgNO_3_ solution turned the solution first light brown and then dark brown. The creation of AgNPs was monitored and confirmed by a single UV absorption peak centered at 425 nm as shown in Figure 3 [40,52]. The formation of ZnONPs was visually confirmed by the color change of the Zn(NO_3_)_2_ solution from off-white to yellowish after the addition of neem leaf extract. A typical peak of ZnONPs was recorded at 378 nm as shown in Figure 3 [53,54,55].

**Figure 4:**
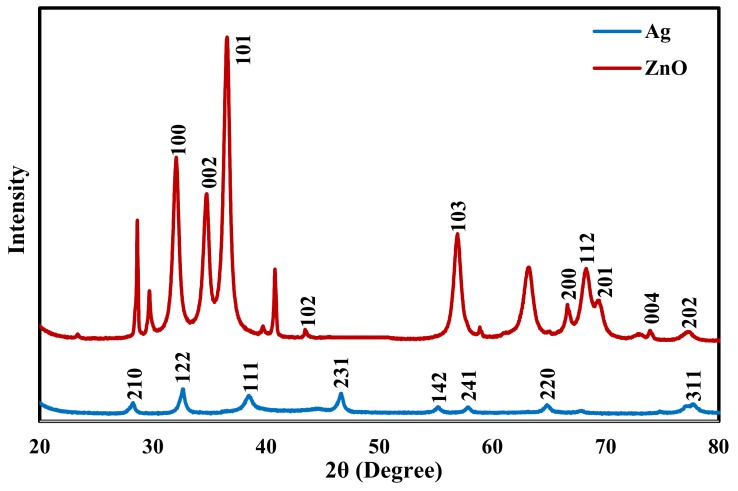
XRD pattern of AgNPs and ZnONPs.

To further confirm the formation of AgNPs and ZnONPs X-ray diffraction patterns of the two samples were collected and are shown in Figure 4. For AgNPs Bragg reflection peaks at 2θ values of 28°, 33°, 38°, 46°, 55°, 57°, 64°, and 77° were observed, which are related to (210), (122), (111), (231), (142), (241), (220) and (311) planes of face-centered cubic silver structure (ICDD 893722) [47]. For ZnONPs, reflection peaks appeared at 2θ of 32°, 34°, 37°, 44°, 56°, 66°, 68°, 69°, 74°, and 77°. These peaks are related to the planes (100), (002), (101), (102), (103), (200), (112), (201), (004), and (202) of the standard ZnO hexagonal wurtzite polycrystalline structure (JCPDS 361451) [7,56].

The XRD results clearly show that the AgNPs and ZnONPs prepared by the Neem extract are both crystalline in nature. The crystallite size of AgNPs and ZnONPs were calculated by the Debye-Scherer’s equation [47]:

(1) $$D=\frac{0.9\lambda }{\beta \cos\theta }$$

where D is the size of the crystal, λ is the applied X-ray wavelength, θ is the Bragg’s angle, and β is the full width of the diffraction peak at half maximum. The crystallite size of obtained Ag was around 6.4 nm based on the (122) reflection, while for ZnO, it was around 7.5 nm and obtained from the (101) reflection.

**Figure 5:**
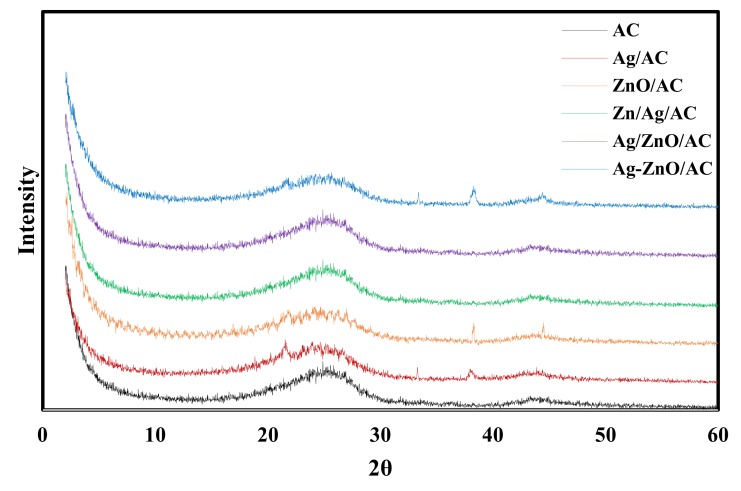
X-ray diffraction of AC, Ag /AC, ZnO/AC, Ag/ZnO/AC, ZnO/Ag/AC, and Ag-ZnO/AC.

The XRD patterns of the five nanocomposites are shown in Figure 5. The pure AC shows two very wide diffraction peaks at 2θ of 24° and 44° which are related to (002) and (100) [9,57]. The Ag/AC pattern shows peaks at 2θ of 33°, 38°, and 45° corresponding to the (122), (111), and (231) crystal planes of the cubic lattice structure of AgNPs. Broad peaks of AC and the sharp peaks of silver appeared in the results of the Ag/AC confirming the successful impregnation of AgNPs on AC surface [9]. Similar observation for Ag/AC XRD pattern was reported by El-Aassar et al. [6] and Tuan et al. [9]. The ZnO/AC pattern shows two wide diffraction peaks at 2θ of 38°and 45° which are related to (101) and (102). The Ag-ZnO/AC pattern displays peaks at 2θ of 33°, 38°and 44° which are related to (122), (111) and (231) of Ag and (101) and (102) for ZnO. Ag/ZnO/AC and ZnO/Ag/AC patterns show almost the same weak peaks at 37°and 44° which are related to (111) and (231) of Ag and (101) and (102) for ZnO.

**Figure 6:**
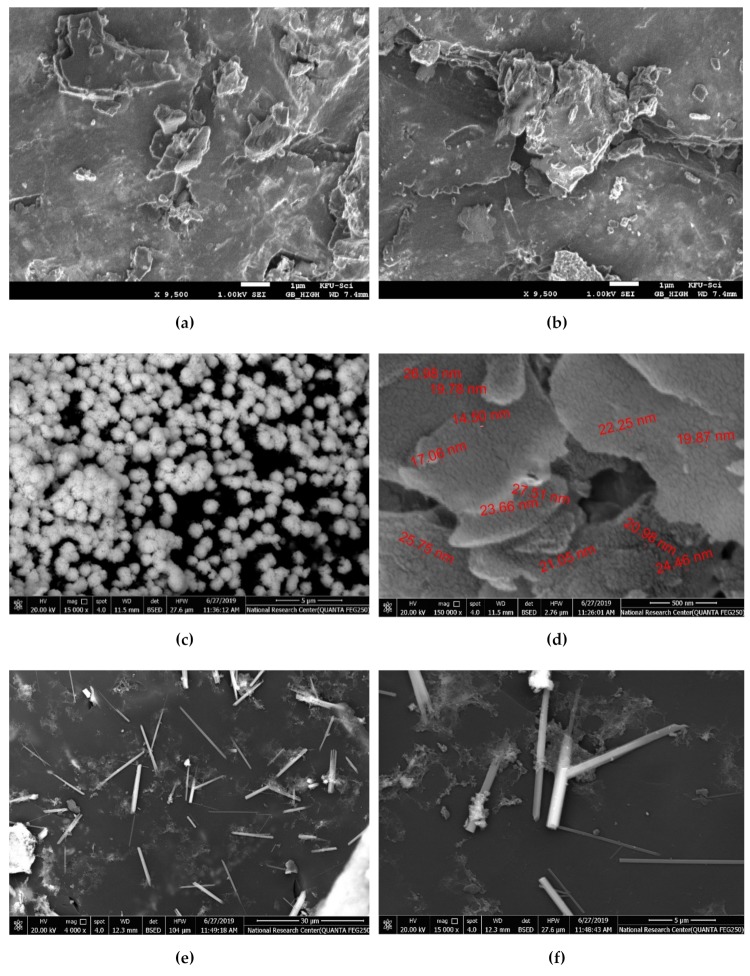

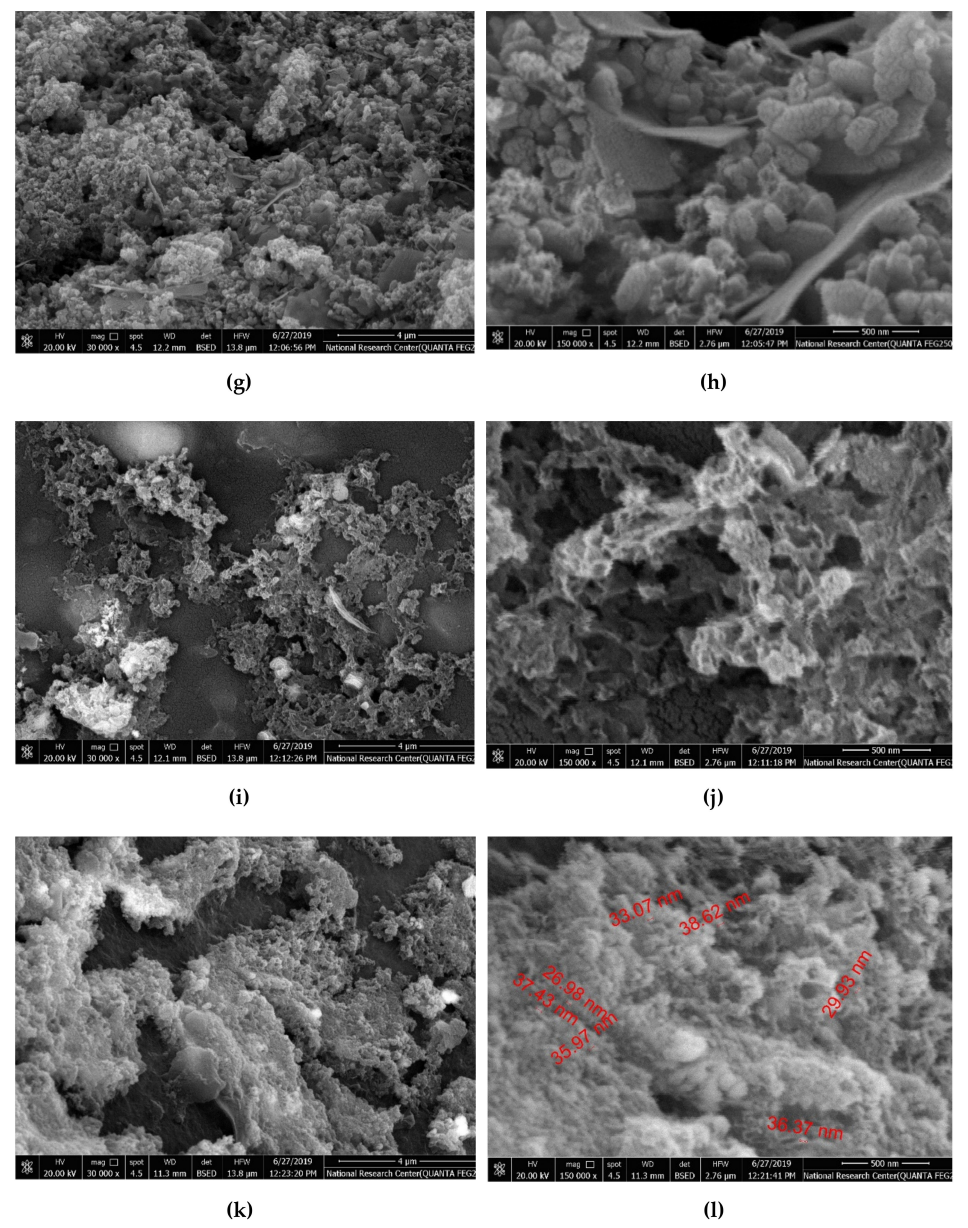
SEM images of bare AC (**a**, **b**), Ag/AC (**c**, **d**), ZnO/AC (**e**, **f**), ZnO/Ag/AC (**g**, **h**) Ag/ZnO/AC (**i**, **j**), and Ag-ZnO/AC (**k**, **l**) at two different magnifications for each sample.

SEM analysis was used to analyze the morphological structure of synthesized nanocomposite. Figure 6a,b show that there was no change in the surface topography of the activated carbon before (a) and after chemical treatment (b). The AC surface is rough with a heterogeneous nature. This rough surface can be easily occupied by AgNPs and ZnONPs. Furthermore, the phenolic, lactonic and carboxylic functional groups of the AC are capable of having physical interactions with the prepared nanoparticles via stabilizing ligands [2]. The AgNPs on AC surface in sample Ag/AC are spherical with an average diameter around 20 nm with some aggregation and good dispersion on the surface as shown in Figure 6c,d. Similar Ag/AC SEM patters were recorded by Tuan et al. [9].

Figure 6e,f show that the green synthesized ZnO/AC predominantly formed nanorod shapes. The ZnO nanorods have a diameter around 300 nm and length of 5 µm. It is clear that there is no aggregation of the nanorods. Lepot et al. reported the synthesis of crystalline one-dimensional ZnO nanorods have diameters of 50–200 nm and lengths up to 5 μm by hydrothermal process with zinc acetate as the precursor [58].

The ZnO/Ag/AC sample formed spherical and flakes like particles with a high degree of aggregation. It is apparent that spherical shapes dominates over flakes as shown in Figure 6g,h. Both the Ag/ZnO/AC and Ag-ZnO/AC samples demonstrated sponge-like structured nanoparticles. In the Ag/ZnO/AC sample a mixture of nanorods and spherical particles were obtained with a high degree of aggregation whiler Ag-ZnO/AC mainly displayed a spherical shape with an average diameter of 35 nm. The bright spots appearing in the SEM images confirm the successful incorporation of AgNPs and ZnONPs on the AC surface [3].

### 2.1. Catalysis Activity of the Prepared Samples

**Figure 7:**
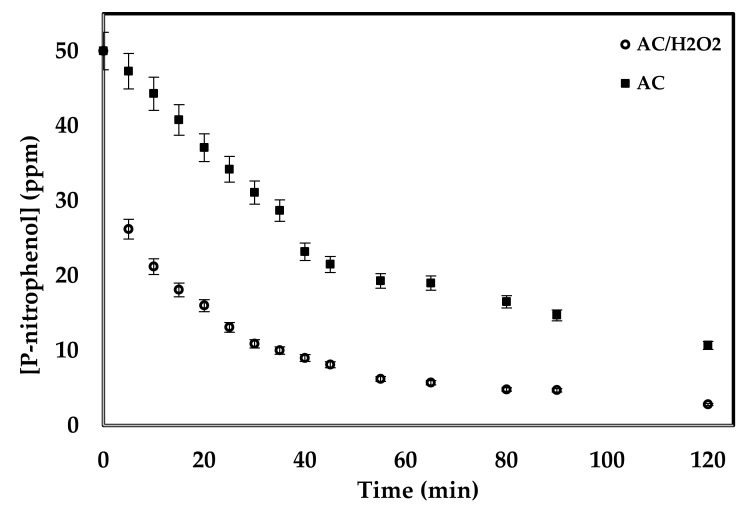
Effect of addition of H_2_O_2_ on the decomposition of 4-nitrophenol under the conditions of 20 mL of 50 ppm 4-nitrophenol, 2 mL of H_2_O_2_, 0.3 g of AC, 25 °C, 250 rpm for 2 h.

The catalytic activity of the prepared samples was tested against the decomposition of 4-nitrophenol in aqueous solution with a concentration of 50 ppm at 25 °C. For comparison purposes, free activated carbon was also tested. To investigate the necessity of adding H_2_O_2_ to the decomposition mixture, two tests were performed using bare AC samples with and without adding H_2_O_2_. Results shown in Figure 7 indicate the important rule of H_2_O_2_ in the decomposition of 4-nitrophenol. When H_2_O_2_ was used, a concentration of about 20 ppm lower than the case without using H_2_O_2_ was achieved. After 2 h of decomposition time, the final concentration of 4-nitrophenol in the presence of H_2_O_2_ was 2.8 ppm compared to 10.7 ppm in the absence of H_2_O_2_. Thus, the rest of the experiments were performed in the presence of H_2_O_2_ [59,60].

**Figure 8:**
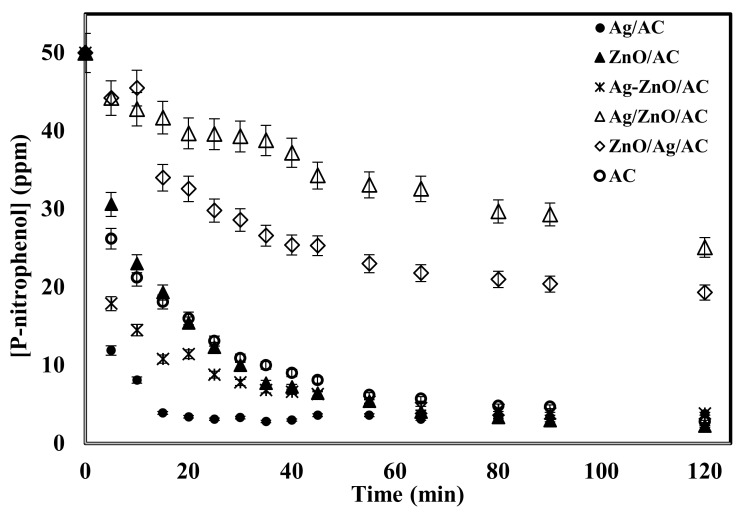
Decomposition of 4-nitrophenol using different catalysts under the conditions of 20 mL of 50 ppm 4-nitrophenol, 2 mL of H_2_O_2_, 0.3 g of catalyst, 25°C, 250 rpm for 2h.

Figure 8 shows that the two samples prepared by successive precipitation of ZnO and Ag on the AC surface (ZnO/Ag/AC and Ag/ZnO/AC) have lower catalytic activity compared to bare AC, perhaps due to nanoparticle aggregation and low dispersion of the nanoparticles as shown in the SEM images of theses samples. On the other hand, the sample prepared by co-precipitation of both Ag and ZnO (Ag-ZnO/AC) showed a better catalytic activity during the first 50 min of decomposition and almost resulted in the same final 4-nitrophenol concentration after 2 h. The sample prepared by precipitation of only ZnO (ZnO/AC) almost has the same performance as the bare AC indicating that ZnO has no catalytic activity for the reduction of 4-nitrophenol. The sample with the best performance was the sample prepared by precipitation of Ag only (Ag/AC). Figure 8 shows that the 4-nitrophenol concentration dropped to 3.4 ppm after only 20 min of reaction. This mainly related to small particles size and good distribution on the AC surface as confirmed by the SEM images.

The reduction reaction of 4-nitrophenol was further tested with both AgNPs and ZnONPs. It is known that in alkaline medium, the absorption peak at 317 nm for 4-nitrophenol will be shifted to 400 nm due to the formation of nitrophenolate ions [23,24,61]. Therefore, the conversion of 4-nitrophenol to nitrophenolate ions catalyzed by AgNPs and ZnONPs can be tested by following the change of absorption peak of nitrophenolate ions at 400 nm, and that of 4-aminophenol at 317 nm [24].

**Figure 9:**
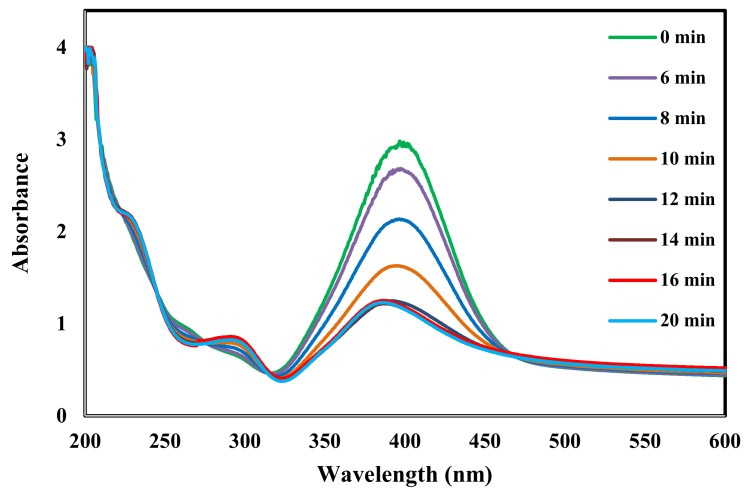
Time-dependent UV-vis spectra for monitoring 4-nitrophenol reduction by catalyzed by AgNPs under the conditions of 20 mL of 50 ppm 4-nitrophenol, 2 mL of H_2_O_2_, 0.3 g of Catalyst, 25 °C, 250 rpm for 20 min.

**Figure 10:**
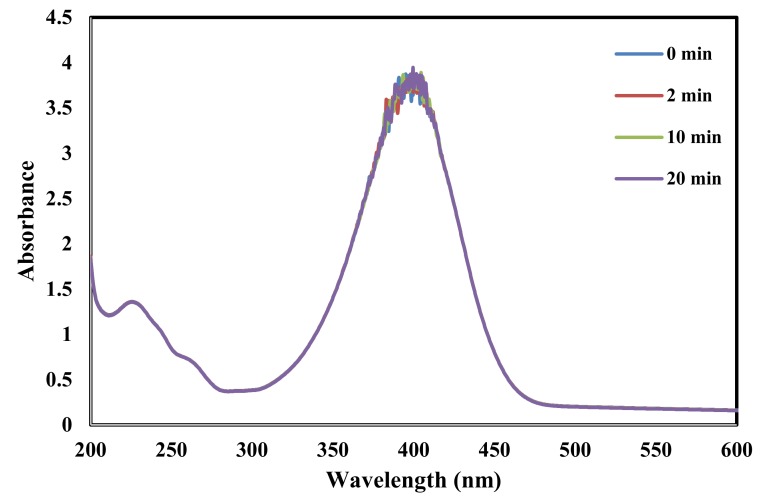
Time-dependent UV–vis spectra for monitoring 4-nitrophenol reduction by catalyzed by ZnONPs under the conditions of 20 mL of 50 ppm 4-nitrophenol, 2 mL of H_2_O_2_, 0.3 g of Catalyst, 25 °C, 250 rpm for 20 min.

Figure 9 and Figure 10 show the time-dependent UV-visible absorption spectra for the reduction reaction in the presence of AgNPs and ZnONPs, respectively. After the addition of AgNPs, Figure 9 shows that the peak at 400 nm due to nitrophenolate ions decreased in intensity while a new peak at 300 nm appeared due to the formation of 4-aminophenol [37,61]. Sudhakar and Soni synthesized silver nanoparticles loaded onto AC using NaBH_4_. Their AgNPs mean particle size was in the range 20–40 nm. They applied it for the reduction of 4-nitrophenols to aminophenols. They found that the reduction was complete in 5 min [24]. On the other hand, Figure 10 shows no change in the UV spectrum for the same time interval when the ZnONPs catalyst used indicating that ZnO had no catalytic activity in this reaction.

The formation of hydroxyl radicals (OH-) during the catalytic process over ZnONPs is shown in Equations (2) and (3) [21]. However, it is known that ZnONPs has a high recombination rate of photo-induced hole-electron pairs produced in reaction 2, and thus reaction 3 will not be achieved and this hinders ZnONPs catalytic activity [27,28].

(2) $$\mathrm{ZnO}+\mathrm{hv}\;\to \mathrm{ZnO}\left(e^{-}+{\;h}^{+}\right)$$

(3) $$h^{+}+{\;H}_{2}O\to H^{+}+{\mathrm{OH}}^{-}$$

### 2.2. Antibacterial Activity

Table 1 shows the zones of inhibition (in mm) obtained for tested clinical strains. It shows clearly a significant activity for Ag-ZnO/AC for both Gram bacteria classes, with ability to inhibit the growth of *E. coli*, *A. baumanni*, *P. aeruginosa*, *K. pseudomonas*, and *S. aureus*. Similarly, Ag-ZnO/AC was more effective against *P. aeruginosa*, *K. pseudomonas* and *S. aureus*. On the other hand, Ag-ZnO/AC seems to be not active against *E. cloacae*. Of three nanostructures designed by Kumar et al., only Ag/ZnO-AC nanohybrid showed an antimicrobial activity against *E. coli*, *P. aeruginosa* and *S. aueus*. Their results were very similar to those found in the current study with Ag-ZnO/AC prepared by the coprecipitation route. Furthermore, our nanohybrid Ag-ZnO/AC seems to be more efficient on *K. pseudomonas* compared to *E. coli* [57].

The ZnO/AC sample was active against 50% of the tested isolates, especially on *E. coli*, *A. baumanni*, *P. aeruginosa*, *K. pseudomonas*, and *S. aureus*. The highest inhibition zone diameter was observed for both *E. coli* and *S. aureus*. In contrast, the ZnO/AC sample was inactive against *E. cloacae*. Compared to similar study, our sample ZnO/AC was more efficient against *E. coli* (12 mm). [57] Fahimmunisha et al. described the proficient antibacterial properties of ZnONPs against *E. coli* and *K. pseudomonas* as described in this current work [37].

Similar to Ag-ZnO/AC and ZnO/AC, the Ag/ZnO/AC sample was active against *P. aeruginosa* and *S. aureus*. Conversely to all the tested samples, Ag/ZnO/AC was the only component able to inhibit the growth of *E. cloacae*. Few data on antibacterial activities against *E. cloacae* is available in the literature. Studies are typically focused especially on *E. coli*, *K. pseudomonas* and *S. aureus* [3,4,6,57]. Ag/ZnO/AC was the worst of the samples tested based on the measured inhibition zone diameter [4]. Kohsari et al. showed a significant zone of inhibition for *E. coli*, *P. aeruginosa*, *S. aueus* and *A. baumani* MDR strains tested against AgNPs without activated carbon. Compared to Kohsari et al.’s study, our results demonstrate an increased diameter when activated carbon was utilized with AgNPs against *P. aeruginosa* (18 mm) and *S. aueus* (19 mm) whilst antibacterial activity for *A. baumani* (14 mm) was unchangeable [62]. The Ag-ZnO/AC nanostructure seems to be more efficient when prepared using the coprecipitation method compared to nanohybrids with AC. Regarding *E. coli*, the zone of inhibition noted for ATCC was greater than MDR, this may be explained by a possible pump efflux mechanism for ACNPs [62].

The Ag/AC sample was active against *A. baumanni*, *P. aeruginosa* and *S. aureus*. The maximum diameter was obtained for both *P. aeruginosa* and *S. aureus*. Table 2 shows some results reported in the literature using Ag/AC and ZnO/AC nanocomposites. Many studies reported that NPs are almost active against *S. aureus* [37].

Understanding of a possible mechanism of action is usually needed. Many studies report mechanisms of action of common NPs separately such as ZnONPs and AgNPs. Nevertheless, both ZnONPs and AgNPs are known for their great antibacterial activity against Gram class bacteria [3,38,51]. Many studies reported the existence of both amino and carboxyl groups on the cell surface with a high affinity that may justify these antibacterial activities for both Gram bacterial classes. ZnONps and AgNps with decreased particle size leading to an increased surface area along with a good affinity for these groups and exceeded interaction and was also found to have an elevated ability to penetrate cells [37]. ZnONps containing zinc ions and AgNPs containing silver ions act even on DNA, destroying the double helix chain structure. Different mechanisms were reported in the literature about antibacterial activity against different Gram class species as shown in Table 3 [52,63]. Biochemical structure composition for both Gram classes’ cell membranes let different nanoparticles interact contrastively. Opaque peptidoglycan layer of gram-positive cell membrane leads to an arduous nanoparticle penetration, translated in a decreased inhibition zone compared to the gram-negative bacteria [51]. Controversially, thin peptidoglycan layer of gram-negative cell membrane with negative charge bound to positive charge coming from AgNps [31].

## 3. Materials and Methods

### 3.1. Chemicals

All chemical reagents used in this experimental work were of analytical grade. Silver nitrate as a precursor (AgNO_3_, 99%), zinc nitrate (Zn(NO_3_)_2_) and 4-nitrophenol were purchased from Sigma-Aldrich (St. Louis, MO, USA). All solutions were prepared using De-ionized water and were used throughout the experiments. Activated carbon was obtained from the spent home filtration system filters. A detailed description of this process can be found in our earlier contribution [50].

### 3.2. Preparation of the Activated Carbon

Since the activated carbon used as support was collected from the spent home filtration system, it was necessary to do a regeneration process as described in our previous contribution [50].

### 3.3. Preparation of Neem leaf Extract

Neem leaves were collected from the local area Al-Ahasa, Sudia Arabia and was well washed using tap and distilled water to remove any contamination or impurities. 10 g of freshly chopped leaves were boiled with 100 mL of deionized water for 20 min. the extract was filtered using Whatman filter paper, then kept at 4 °C for further use [44].

### 3.4. Preparation of Ag/AC

Neem leaf extract (10 mL) was added to 1 mM silver nitrate solution (AgNO_3_, 100 mL). The color of the solution directly changed to light brown after the addition of the extract, indicating the formation of silver nanoparticles. The solution was kept in a dark chamber overnight at room temperature to reduce the photoactivation of silver nitrate for impregnation on activated carbon. The reduction process of Ag^+1^ to Ag^0^ was confirmed visually by the presence of a brown color. Also, the formation was confirmed by using UV-1800 spectrophotometer (Shimadzu, Kyoto, Japan). Activated carbon (5 g) was added to 100 mL of the as-prepared silver nanoparticles with vigorous stirring overnight at room temperature to ensure the best coating of silver nanoparticles on activated carbon. The Ag/AC sample was extensively washed using deionized water and ethanol and air-dried for further characterization.

### 3.5. Preparation of ZnO/AC

To synthesize ZnO nanoparticles, 10 mL of neem leaf extract was added to a 100 mL solution of 0.05 M (Zn(NO_3_)_2_) under vigorous stirring at 70 °C, followed by the addition of 1 M NaOH solution and adjustment of the pH to 8 resulting in formation of a white-yellow precipitate. Then, 5 g of activated carbon was added and the solution was left overnight with stirring. The ZnO/AC sample was washed with deionized water and ethanol, then it was air-dried for further characterization.

### 3.6. Preparation of ZnO/Ag/AC by Successive Precipitation

Previously prepared ZnO nanoparticles solution (40 mL) was mixed well with 5 g of as-prepared Ag/AC. The mixture was left on a magnetic heating stirrer overnight at 70 °C with vigorous stirring. The ZnO/Ag/AC was filtered and washed very well with double distilled water and then dried at 110 °C for 12 h.

### 3.7. Preparation of Ag/ZnO/AC by Successive Precipitation

As-prepared ZnO/AC (5 g) was mixed with 40 mL of previously prepared AgNPs solution. The mixture was placed on a magnetic heating stirrer overnight. The Ag/ZnO/AC was then filtered off and extensively washed with double distilled water and dried at 110 °C for 12 h.

### 3.8. Preparation of Ag-ZnO/AC by Co-Precipitation

Activated carbon-supported silver nanoparticles and zinc oxide nanoparticles prepared using a co-precipitation method were prepared by mixing 1 mM AgNO_3_ (50 mL) with 0.05 M (Zn(NO_3_)_2_) then 50 mL of neem leaf extract was added, the mixture pH was adjusted to 8 by using 1 M NaOH. Then it was mixed with 10 g of activated carbon in a 400 mL beaker under magnetic stirring with heating to 70 °C to up to 24 h, resulting in the formation of the AgNPs and ZnONPs on the activated carbon (Ag-ZnO/AC). The Ag-ZnO/AC was then filtered and extensively washed with double distilled water. The Ag-ZnO/AC was then dried at 110 °C. All samples identification and preparation methods were described in Table 4.

### 3.9. Catalytic Activity

Photodegradation of 4-nitrophenol was carried out at different intervals of time ranging from 2 min to 3 h at 20 °C. In a 250-mL beaker, 20 mL of 50 ppm 4-nitrophenol solution was mixed with 2 mL of H_2_O_2_ and the pH was adjusted at 8. Then, 20 mg of AC, Ag/AC, ZnO/AC, Ag/ZnO/AC, ZnO/Ag/AC, or Ag-ZnO/AC sample was added to the solution with stirring at 250 rpm and the concentration of 4-nitrophenol was followed by measuring the UV absorbance at λ_max_ of 317 nm.

### 3.10. Anti-Bacterial Activity

A quantitative antimicrobial study was carried on determining the presence of eventual antimicrobial activity. The inhibition zones were measured and compared for each sample. Only samples with significant inhibition zones were collected for further experiments. Primary screening was done using control strain *E. cole* J57 on MHA. Sixteen different clinical isolates of different origins were collected in July 2019 with the collaboration of the College of Medicine of Tunis Laboratory of Resistance to Antimicrobial Agents. Particles were tested against the 13 different Gram-negative and three Gram-positive bacteria shown in Table 1. The microbial identification was determined by Gram staining, oxidase test, and API 20E strips (BioMérieux, Marcy-L’Etoile, France). Antimicrobial susceptibilities for clinical strains were determined by the disk diffusion method based on the inhibition zone, according to the European Committee on Antimicrobial Susceptibility Testing recommendations (CA-SFM/EUCAST, 2016). All chosen strains were 100% resistant to 3rd generation cephalosporin (C3GR). In the same way, *Pseudomonas aeruginosa* was resistant also to both C3G and conversely to carbapenems. Antimicrobial activities were determined by the diffusion method. Plates containing MHA were perforated using sterile tips with 6 mm of diameters. The samples tested were Ag/AC, ZnO/AC, Ag/ZnO/AC, ZnO/Ag/AC, and Ag-ZnO/AC. A quantity of 0.12 g was introduced in sterile conditions directly on holes created on the medium. All dishes were incubated overnight for 24 h at 37 °C. The inhibition zones were measured and compared with standard isolates and sample controls.

### 3.11. Characterization

Absorption studies were recorded with a UV-1800 spectrophotometer (Shimadzu, Kyoto, Japan). The surface morphology and shape were investigated by Scanning Electron Microscopy (SEM) using a JSM-7600F high-resolution field instrument (JEOL, Akishima, Tokyo, Japan). FT-IR spectra of samples were collected using a 630 FT-IR spectrophotometer (Cary, South San Francisco, CA, USA). X-ray diffraction (XRD) analysis was conducted using an XRD 3100 diffractometer (Philips, Amsterdam, The Netherlands) at 45 kV and 30 mA. Copper Kα radiation and a graphite monochromator to produce X-rays with a wavelength of 1.54060 Å were used. Samples were placed in a glass holder and scanned from 10° to 60° with a scanning rate of 2.0°/min [33].

## 4. Conclusions

In this current study, an eco-friendly, cost effective, simple, rapid, and green approach was successfully applied to the synthesis of different nanocomposites of AgNPs and ZnONPs loaded on regenerated activated carbon using the neem leaf extract for the reduction and stabilization of the nanoparticles. Among the prepared samples, the Ag/AC and Ag-ZnO/AC nanocomposites have shown a very promising catalytic degradation activity toward 4-nitrophenol. Furthermore, these samples showed a significant antibacterial activity against both bacterial Gram classes. This environmentally friendly approach could compete with the conventional routes of synthesis in terms of cost, safety, simplicity, and effectiveness and thus has the potential to be used in water treatment in addition to the drug industry.

## Figures and Tables

**Table 1:** Antimicrobial activity based on the inhibition zone diameter for gram-negative and gram-positive bacteria resistant to C3G. Standard deviation ranging from ±0.1 and ±0.86 (*n* = 3).

Strains			Inhibition Zone (mm)				
			Ag/AC	ZnO/AC	Ag/ZnO/AC	ZnO/Ag/AC	Ag-ZnO/AC
Gram-	*Esherichia coli*	ATCC 25983	6	12	6	8	8
		ATCC 29212	6	6	6	6	8
		37420	6	12	6	10	6
	*Acinetobacter baumanni*	4179	10	6	6	6	8
		12594	14	6	6	6	9
		12895	14	8	6	6	8
		22497	6	6	6	6	8
	*Pseudomonas aeruginosa*	1314a	18	8	6	6	12
		12898	12	6	8	6	8
		9381	12	8	8	6	8
	*Klebsiella pseudomonas*	37591	6	10	6	10	12
		22210	6	6	6	10	11
	*Enterobacter cloacae*	37444	6	6	6	14	6
Gram+	*Staphyloccocus aureus*	16678	19	13	18	12	12
		18226	16	8	8	6	11
		29213	6	6	6	6	8

**Table 2:** List of antibacterial activity of similar samples reported in the literature.

Nanocomposite	Pathogens	Inhibition Zone (mm)	Reference
Ag/AC	*E. coli*	18	[3]
Ag/AC	*S. aureus*	11	[57]
	*B. subtilis*	10	
	*P. aeruginosa*	13	
	*E. coli*	10	
ZnO/AC	*S. aureus*	10	[57]
	*B. subtilis*	11	
	*P. aeruginosa*	10	
	*E. coli*	10	
Ag/ZnO/AC	*S. aureus*	14	[57]
	*B. subtilis*	11	
	*P. aeruginosa*	16	
	*E. coli*	14	
Ag/AC	*E. coli*	9–14	[6]
Ag/AC	*E. coli*	Effective (no zone reported)	[4]

**Table 3:** Antibacterial activity screening strains used with ZnONPs and AgNPs and the main mechanisms of action.[37,57].

Gram Class	Bacteria Used in This Study	Strains Screened	Main Mechanism of Action
Gram−	*Esherichia coli*	ATCC 25983 ATCC 29212 37420	Alteration of membrane permeability and respiration.
	*Klebsiella pseudomonas*	37591 22210	Alteration of cell wall.
	*Pseudomonas aeruginosa*	1314a 12898 9381	Alteration of cell wall.
	*Acinetobacter baumanni*	4179 12594 12895 22497	
	*Enterobacter cloacae*	37444	
Gram+	*Staphyloccocus aureus*	16678 18226 29213	Inhibition of replication.

**Table 4:** Sample description and preparation method.

Sample	Composition	Method
Ag/AC	100% AgNPs on AC	Impregnation
ZnO/AC	100% ZnONPs on AC	Impregnation
Ag/ZnO/AC	50% Ag and 50% ZnO on AC	Successive precipitation
ZnO/Ag/AC	50% ZnO and 50% Ag on AC	Successive precipitation
Ag-ZnO/AC	50% Ag and 50% ZnO on AC	Co-precipitation
